# Differences in gene expression in whitefly associated with CYSDV-infected and virus-free melon, and comparison with expression in whiteflies fed on ToCV- and TYLCV-infected tomato

**DOI:** 10.1186/s12864-019-5999-0

**Published:** 2019-08-15

**Authors:** Navneet Kaur, Wenbo Chen, Zhangjun Fei, William M. Wintermantel

**Affiliations:** 1 USDA-ARS, Crop Improvement and Protection Research, 1636 East Alisal Street, Salinas, CA 93905 USA; 2 Boyce Thompson Institute, 533 Tower Road, Ithaca, New York 14853-1801 USA; 3 USDA-ARS, Robert W. Holley Center for Agriculture and Health, 538 Tower Road, Ithaca, New York 14853-2901 USA; 4 Present Address: Driscoll’s Inc., 151 Silliman Rd., Watsonville, CA 95076 USA

**Keywords:** Whitefly, *Bemisia tabaci* MEAM1 (biotype B), *Cucurbit yellow stunting disorder virus*, Crinivirus, RNA-Seq, RNAi, Semipersistent transmission, Arthropod genomics, Orphan genes, Gene expression

## Abstract

**Background:**

*Cucurbit yellow stunting disorder virus* (CYSDV; genus *Crinivirus, Closteroviridae*) is transmitted in a semipersistent manner by the whitefly, *Bemisia tabaci*, and is efficiently transmitted by the widely prevalent *B. tabaci* cryptic species, MEAM1. In this study, we compared transcriptome profiles of *B. tabaci* MEAM1, after 24 h, 72 h and 7 days of acquisition feeding on melon plants infected with CYSDV (CYSDV-whiteflies) with those fed on virus-free melon, using RNA-Seq technology. We also compared transcriptome profiles with whiteflies fed on tomato plants separately infected with *Tomato chlorosis virus* (ToCV), a crinivirus closely related to CYSDV, and *Tomato yellow leaf curl virus* (TYLCV), a member of the genus *Begomovirus*, which has a distinctly different mode of transmission and their respective virus-free controls, to find common gene expression changes among viruliferous whiteflies feeding on different host plants infected with distinct (TYLCV) and related (CYSDV and ToCV) viruses.

**Results:**

A total of 275 differentially expressed genes (DEGs) were identified in CYSDV-whiteflies, with 3 DEGs at 24 h, 221 DEGs at 72 h, and 51 DEGs at 7 days of virus acquisition. Changes in genes encoding orphan genes (54 genes), phosphatidylethanolamine-binding proteins (PEBP) (20 genes), and AAA-ATPase domain containing proteins (10 genes) were associated with the 72 h time point. Several more orphan genes (20 genes) were differentially expressed at 7 days. A total of 59 common DEGs were found between CYSDV-whiteflies and ToCV-whiteflies, which included 20 orphan genes and 6 lysosomal genes. A comparison of DEGs across the three different virus-host systems revealed 14 common DEGs, among which, eight showed similar and significant up-regulation in CYSDV-whiteflies at 72 h and TYLCV-whiteflies at 24 h, while down-regulation of the same genes was observed in ToCV-whiteflies at 72 h.

**Conclusions:**

Dynamic gene expression changes occurred in CYSDV-whiteflies after 72 h feeding, with decreased gene expression changes associated with 7 days of CYSDV acquisition. Similarities in gene expression changes among CYSDV-whiteflies, ToCV-whiteflies and TYLCV-whiteflies suggest the possible involvement of common genes or pathways for virus acquisition and transmission by whiteflies, even for viruses with distinctly different modes of transmission.

**Electronic supplementary material:**

The online version of this article (10.1186/s12864-019-5999-0) contains supplementary material, which is available to authorized users.

## Background

*Cucurbit yellow stunting disorder virus* (CYSDV; genus *Crinivirus*, family *Closteroviridae*), is widely distributed among cucurbit production regions. The virus was initially discovered in the United Arab Emirates [1], but has spread throughout many tropical and subtropical cucurbit production regions of the world, including the Middle East and Mediterranean basin, as well as Central America and southern and western regions of the United States where its vector is prevalent [2]. CYSDV is transmitted in a semipersistent manner by the whitefly, *Bemisia tabaci* [3], and is efficiently transmitted by both of the widely prevalent *B. tabaci* cryptic species, MEAM1 and MED (formerly known as Biotypes B and Q, respectively). Although CYSDV can be acquired by whiteflies from melon leaves with feeding periods as short as two hours, efficient virus acquisition for maximum transmission efficiency requires at 18–24 h and the virus can be transmitted for at least 7 days after removal from virus-infected plants with a gradual decrease in transmission efficiency over that time [3]. Cucurbit plants infected with CYSDV develop striking interveinal chlorosis symptoms, with major veins remaining green until late in disease development [2–4]. Recent studies have demonstrated that infected reservoir host plants vary in their capacity to serve as acquisition hosts for the whitefly vector, with acquisition and transmission of CYSDV more efficient with some host plants (such as cucurbits) than others (such as many common weeds or non-cucurbit host plants) [5, 6]. During virus acquisition by the whitefly vector, CYSDV is believed to associate with the anterior foregut of the whitefly through an association of the virus encoded minor coat protein (CPm) with as yet undetermined components of the insect. This is based on studies conducted on *Lettuce infectious yellows virus* (LIYV), another member of the genus *Crinivirus* [7–9].

Previous studies by our group compared gene expression in whiteflies in response to feeding on tomato plants infected with each of two viruses, *Tomato chlorosis virus* (ToCV; genus *Crinivirus*, family Closteroviridae) and *Tomato yellow leaf curl virus* (TYLCV; genus *Begomovirus*, family Geminiviridae). Although both viruses are considered phloem limited in their host plants, the two viruses have very different modes of transmission by *B. tabaci*. TYCLV is transmitted in a persistent, circulative manner and the virus is retained essentially for the life of the whitefly [10]. In contrast, criniviruses such as ToCV and CYSDV, are transmitted in a semipersistant, noncirculative manner and retained for periods of one to a few days after acquisition, likely in the anterior foregut of the whitefly [7–9], with retention time dependent on the virus and cryptic species (biotype) of the whitefly vector [6, 11]. In studies, conducted in parallel under identical conditions, it was determined that some common changes in gene expression occurred due to the feeding of *B. tabaci* MEAM1 on virus infected tomato plants for the same time periods, even though the two viruses have very different modes of transmission by the whitefly [12, 13]. More specifically, 28 differentially expressed genes (DEGs) were common between whiteflies fed on ToCV-infected tomato vs. virus-free tomato and those fed on TYLCV-infected tomato vs. virus-free tomato [12, 13]. Most of the genes with common altered expression patterns were associated with metabolism [13]. In order to further explore the gene expression changes in whitefly that are important for transmission of criniviruses, we chose to examine the effect of whitefly feeding on a different host plant infected by a distinct but related crinivirus. CYSDV was selected because it is also transmitted efficiently by *B. tabaci* MEAM1, and is relatively closely related to ToCV compared with other members of the genus [14–16]. Finally, it was noted that a shift in gene expression occurred in whiteflies that fed on ToCV at approximately the same time most virus retention ceases in the vector, which occurs between two and three days following virus acquisition for *B. tabaci* MEAM1 [11, 12]. CYSDV has a much longer retention time in *B. tabaci* MEAM1 than does ToCV, remaining transmissible by this vector for seven days following acquisition [3]. Therefore, we evaluated gene expression in whiteflies after feeding on virus-free and CYSDV-infected melon plants for 24 h, 72 h, and 7 days to highlight what the two previous studies suggested would be the most relevant time points for comparative evaluation among the three virus-host pairs. Using two different host plants, tomato and melon, and the closely related criniviruses, ToCV and CYSDV, we identified 59 DEGs in common between whiteflies fed on plants individually infected with ToCV and CYSDV. Furthermore, we found 14 DEGs in common among whiteflies fed on plants infected individually with ToCV, CYSDV, and TYLCV compared with whiteflies fed on the respective virus-free plants. By examining transmission of a second crinivirus from a different host plant, as well as a virus having a longer retention time in the whitefly vector, we gained new insights into vector gene expression associated with transmission of a semipersistant virus and broadened the overall knowledge base of how whiteflies are influenced by feeding on virus infected plants and how gene expression may influence vector transmission efficiency.

## Results

### DEGs in whiteflies associated with feeding on CYSDV-infected melon plants

To determine how global gene expression is differentially influenced in the whitefly when feeding on CYSDV infected melon plants as opposed to healthy melon plants, RNA-Seq was used to compare gene expression in whiteflies that had been fed on CYSDV-infected (‘CYSDV whiteflies’) or uninfected melon plants (‘virus-free [VF] whiteflies’) for acquisition access periods (AAP) of 24 h, 72 h, and 7 d. A total of 11.5–14.9 M raw reads were generated per library. These were processed to remove adapters, low quality reads, and reads from the endosymbionts (*Hamiltonella*, *Rickettsia, and Portiera*), as well as rRNA and mtDNA. This resulted in 10.7–13.9 M cleaned reads per library, of which 82–88% of the reads mapped to the reference genome of the whitefly (*Bemisia tabaci* MEAM1) [17] (Additional file 1a). Pearson’s correlation coefficients analysis showed highly reproducible data across the different replications (Additional file 1b).

**Fig. 1:**
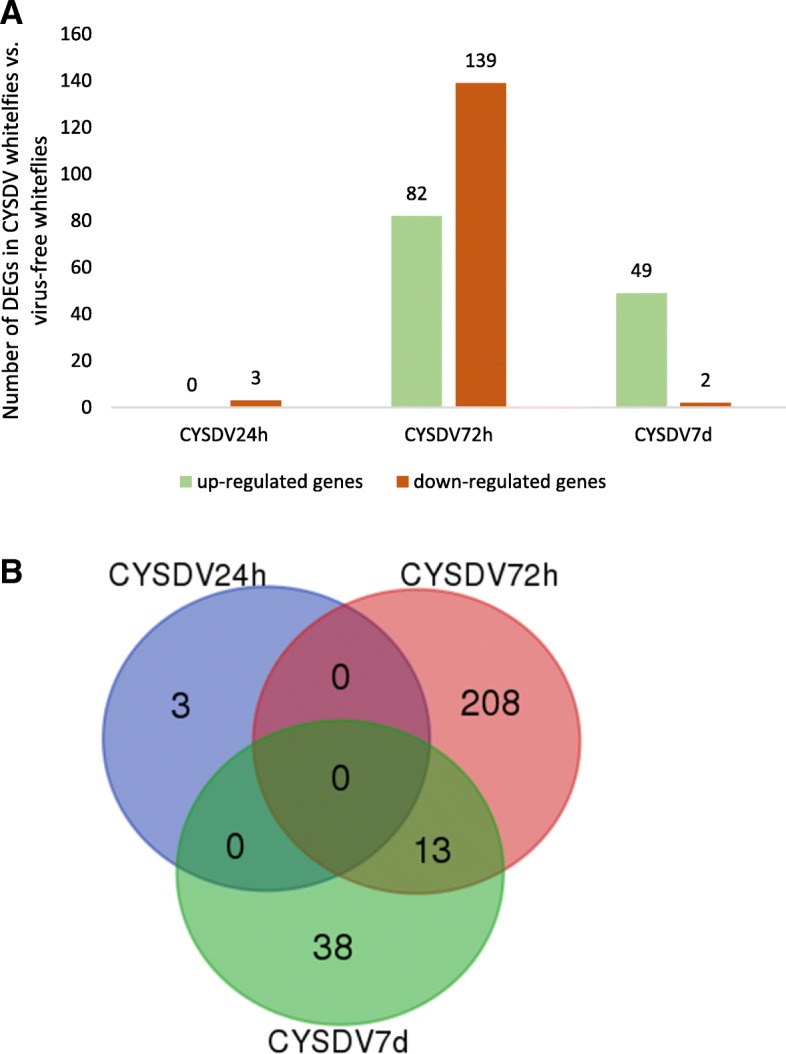
Differentially expressed genes (DEGs) in whitefly, *Bemisia tabaci* MEAM1 following feeding on CYSDV-infected (CYSDV whiteflies) or uninfected (virus-free whiteflies) melon plants for 24 h, 72 h, and 7 d. **a** Number of DEGs detected between CYSDV whiteflies and virus-free whiteflies at three different time points of feeding. **b** Venn-diagram showing unique and common DEGs in whitefly after feeding on CYSDV-infected or uninfected melon plants for three different feeding periods

The whitefly genome contains 15,664 predicted genes, of which 275 were differentially expressed genes (DEGs) in CYSDV whiteflies compared with VF whiteflies at three different AAPs. Three DEGs (all down-regulated) were found after the 24 h AAP, 221 DEGs (82 up-regulated and 139 down-regulated) after the 72 h AAP, and 51 DEGs (49 up-regulated and 2 down-regulated) were present after the 7 d AAP (Fig. 1a, Additional file 1c). The majority of DEGs identified in CYSDV whiteflies were unique to each feeding period; however, some DEGs were common to more than one feeding period (Fig. 1b).

### Virus titers in whiteflies

As noted previously, feeding periods of 18 to 24 h provide sufficient virus acquisition to maximize transmission efficiency and retention of initially acquired virus will decline gradually over a period of seven days [3]; however, continued feeding on an infected source as conducted during these experiments should keep virus levels high in whiteflies throughout the acquisition feeding period. To evaluate virus levels in whiteflies we conducted RT-qPCR on whiteflies at the two later time points (72 h and 7 days), the two time points at which large numbers of DEGs were identified between virus-free and CYSDV whiteflies. It was interesting that the seven-day feeding period still allowed accumulation of more virus (2.3 × 10^4^ copies/ug) than was found after the 72 h feeding period (5.2 × 10^3^ copies/ug) suggesting that virus binding in the whitefly may not saturate all sites even after several days of feeding. However, it is equally likely that differences in virus levels within whiteflies between the two virus acquisition access periods could be related to greater ingestion of virus into whiteflies after the 7 d acquisition period rather than the amount of virus available for transmission from binding sites in the anterior foregut.

### DEGs at the 24 h feeding time point

In this study, CYSDV was selected for the RNA-Seq analysis due to its ability to be transmitted by *B. tabaci* MEAM1 like ToCV; however, its retention time in the whitefly vector can last up to 7 days following acquisition [3], which is much longer than that for ToCV [11]. In order to evaluate gene expression changes, time points were selected that were comparable to those used with ToCV, as well as for evaluation of a period corresponding to the end of the longer CYSDV retention period in case gene expression differences may correlate with virus retention. Therefore, sampling was performed after 24 h, 72 h, and 7 d AAPs.

Following the 24 h AAP, only three genes exhibited differential regulation between CYSDV whiteflies and VF whiteflies. All three genes were down-regulated: *Bta03957* (Collagen alpha-1(XII) chain), *Bta03959* (Matrilin-2), and *Bta14954* (Serine protease) (Additional file 1c).

### DEGs at the 72 h feeding time point

Among the 82 genes that were up-regulated in CYSDV whiteflies compared to VF whiteflies following the 72 h AAP, 21 were classified as orphan genes, because they did not show any homology to known proteins. Another major gene category showing up-regulation after the 72 h AAP consisted of five cuticle proteins (*Bta07301*, cuticle protein 16.5-like; *Bta02583*, cuticle protein 7; *Bta07720*, cuticle protein 19; *Bta08284*, cuticle protein 6; *Bta14107*, cuticle protein 1) (Table 1). One of these cuticle proteins, Bta14107, also showed low levels of up-regulation in ToCV whiteflies during previous studies (Table 1) [12]. DEGs with fragments per kilobase of transcript per million reads mapped (FPKM) > 10 and fold change (FC) > 2 are highlighted because we hypothesized that these might be more likely to have important roles in whitefly-CYSDV interactions than other DEGs with lower FPKM values. Those DEGs were: cuticle protein 16.5 (*Bta07301*), cuticle protein 1 (*Bta14107*), three orphan genes (*Bta07683*, *Bta01566*, and *Bta11224*), and one muscle specific protein 300 kDa, isoform I (*Bta00243*) (Table 2).

**Table 1:** Cuticle proteins exhibiting differential expression in viruliferous whiteflies compared to virus-free whiteflies when fed on CYSDV-infected melon and ToCV-infected tomato plants

		FC^a^ (CYSDV/ VF-WF)		FC^b^ (ToCV/ VF-WF)
Gene ID	Annotation	72 h	7 d	24 h
Bta07301	Cuticle protein 16.5-like	16.3	18.38	*
Bta02583	Cuticle protein 7	10.06	9.86	*
Bta07720	Cuticle protein 19	9.3	15.43	*
Bta08284	Cuticle protein 6	6.29	7.77	*
Bta14107	Cuticle protein 1	3.2	3.42	2.05
Bta00254	Cuticular protein 73D	*	3.46	*
Bta09570	Cuticle protein	*	*	0.04
Bta09574	Cuticle protein	*	*	0.04
Bta09553	Cuticle-like protein-2	*	*	0.08

All DEGs have FDR < 0.05

^a^Fold change (FC) values for cuticle proteins in CYSDV vs. virus-free (VF) whiteflies at 72 h and 7 d

^b^FC values for cuticle proteins in ToCV vs. virus-free whiteflies at 24 h

*FC values not significant

**Table 2:** Top differentially expressed genes (DEGs) in whitefly (*B. tabaci* MEAM1) in response to feeding on CYSDV-infected or virus-free (VF) melon plants for 72 h (A) and 7 days (B)

A					
Gene ID	Annotation	VF72^a^	CYSDV72^b^	FC^c^	FDR^d^
Bta07301	Cuticle protein 16.5 like	0.65	10.61	16.3	8.26E-05
Bta07683	Unknown protein	1.92	13.56	7.06	3.24E-03
Bta14107	Cuticle protein 1	3.44	11.01	3.2	1.68E-03
Bta01566	Unknown protein	5.24	15.71	3	8.06E-03
Bta11224	Unknown protein	167.14	452.74	2.71	8.32E-08
Bta00243	Muscle-specific protein 300 kDa, isoform I	23.22	47.15	2.03	3.60E-03
Bta14312	Sucrase	12.70	5.13	0.4	1.40E-03
Bta10448	Unknown protein	31.07	12.95	0.42	2.72E-02
Bta13678	Unknown protein	20.90	9.06	0.43	6.28E-03
Bta14422	Alpha-glucosidase	20.00	8.82	0.44	4.59E-10
Bta05871	von Willebrand factor A domain-containing protein 2	20.17	9.43	0.47	1.31E-02
Bta07312	Ammonium transporter, putative	35.53	17.45	0.49	1.27E-06
B					
Gene ID	Annotation	VF7d^a^	CYSDV7d^b^	FC^c^	FDR
Bta07301	Cuticle protein 16.5 like	0.71	13.01	18.38	8.62E-04
Bta07683	Unknown protein	1.34	15.72	11.7	8.00E-04
Bta01566	Unknown protein	3.22	22.48	6.99	7.85E-04
Bta14107	Cuticle protein 1	3.76	12.88	3.42	1.20E-02
Bta11224	Unknown protein	97.30	277.91	2.86	3.23E-06
Bta01518	Unknown protein	5.88	13.43	2.28	1.20E-02
Bta12169	Unknown protein	5.15	11.70	2.27	4.22E-02
Bta08648	Dumpy, isoform AA	5.49	12.14	2.21	1.91E-03
Bta15753	Unknown protein	20.55	45.16	2.2	1.60E-03
Bta13640	Chemosensory protein	27.97	61.04	2.18	2.46E-02

DEGs were selected based on criteria of FPKM> 10 with FC > 2 for up-regulated DEGs and FC < 0.5 for down-regulated DEGs

^a^FPKM values from virus-free (VF) whiteflies at 72 h

^b^FPKM values from CYSDV whiteflies at 72 h

^c^Fold change (FC) for CYSDV vs. virus-free (VF) whiteflies

^d^False Discovery Rate (FDR) for three technical replicates of each of three biological replicates

A vastly greater number of down-regulated genes were found in whiteflies after feeding on CYSDV-infected melon for the 72 h AAP compared to the 24 h AAP. Among the 139 DEGs in CYSDV whiteflies showing down regulation compared to VF whiteflies after the 72 h feeding period, five major categories of DEGs were identified: orphan genes (33 genes), phosphatidylethanolamine-binding protein (PEBP) (20 genes), AAA-ATPase domain containing protein (10 genes), dynein heavy chain 3, 7, 5, 10 (four genes), cathepsins 2B, and F-like, L (four genes) (Additional file 1c).

### DEGs at 7 d feeding time point

Only 51 genes (49 up-regulated and 2 down-regulated) showed significant differential regulation in CYSDV whiteflies compared with VF whiteflies after the 7d AAP (Additional file 1c). Three major up-regulated gene categories were identified, and included: 20 orphan genes, six cuticle proteins, and four myosin proteins (myosin regulatory light chain 2, myosin-7-like protein, myosin 2 light chain, and myosin-9, putative).

As described above the first two gene categories, orphan genes and cuticle proteins, were also differentially regulated in CYSDV whiteflies compared with VF whiteflies after the 72 h feeding period. Only five (*Bta11224*, *Bta07683*, *Bta01754*, *Bta15753*, and *Bta01566*) out of a total of 54 orphan genes that were differentially expressed between CYSDV- and VF-whiteflies were expressed differentially at both 72 h and 7 d time points (Table 2). Additionally, five cuticle genes (*Bta07301*, *Bta02583*, *Bta07720*, *Bta08284*, and *Bta14107*) out of the six cuticle genes that were up-regulated in CYSDV whiteflies after the 7 day AAP were also found to be up-regulated in CYSDV whiteflies after the 72 h AAP compared to VF whiteflies (Table 1).

Among the ten most DEGs with FPKM> 10 and FC > 2 with significant *p*-values that were up-regulated in CYSDV whiteflies at 7 days, six were orphan genes, two were cuticle proteins 70 and 1, one dumpy isoform AA, and one chemosensory protein (Table 1b). No genes were found to be down-regulated using the same criteria in CYSDV whiteflies compared to VF whiteflies at the 7 days feeding time point.

### KEGG pathways analysis

**Fig. 2:**
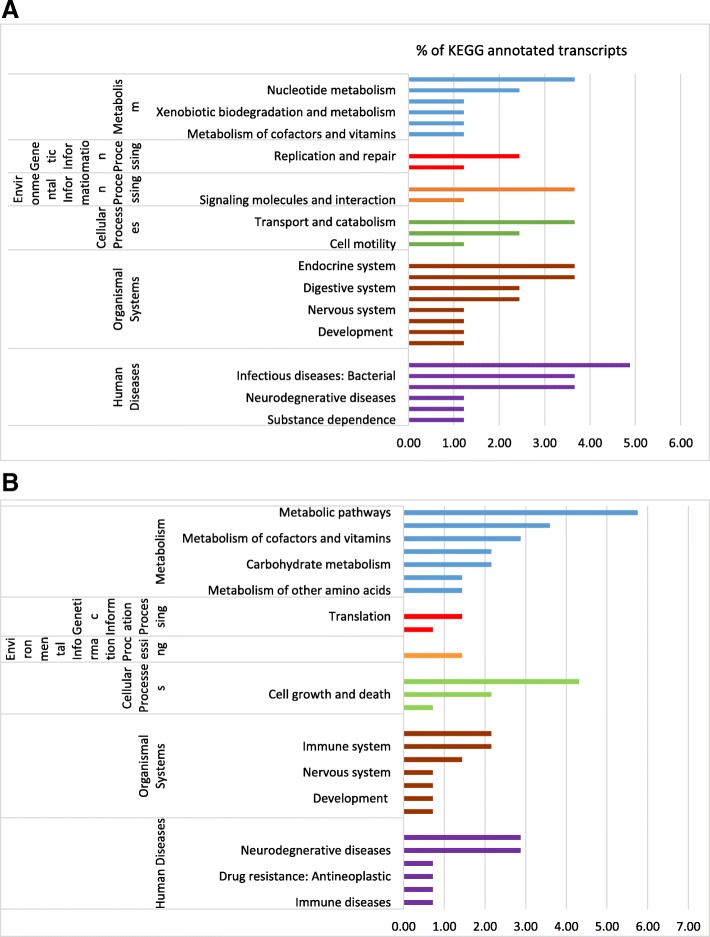
Summary of the KEGG reference pathways associated with up-regulated (**a**) and down-regulated genes (**b**) from whiteflies fed on CYSDV-infected melon (CYSDV whiteflies) for 72 h. Bars represent the percentage of the total KEGG annotated transcripts (28 genes out of a total of 82 up-regulated genes and 40 genes out of a total of 139 down-regulated genes) that mapped to KEGG pathways in the CYSDV whiteflies after 72 h compared to virus-free whiteflies

**Fig. 3:**
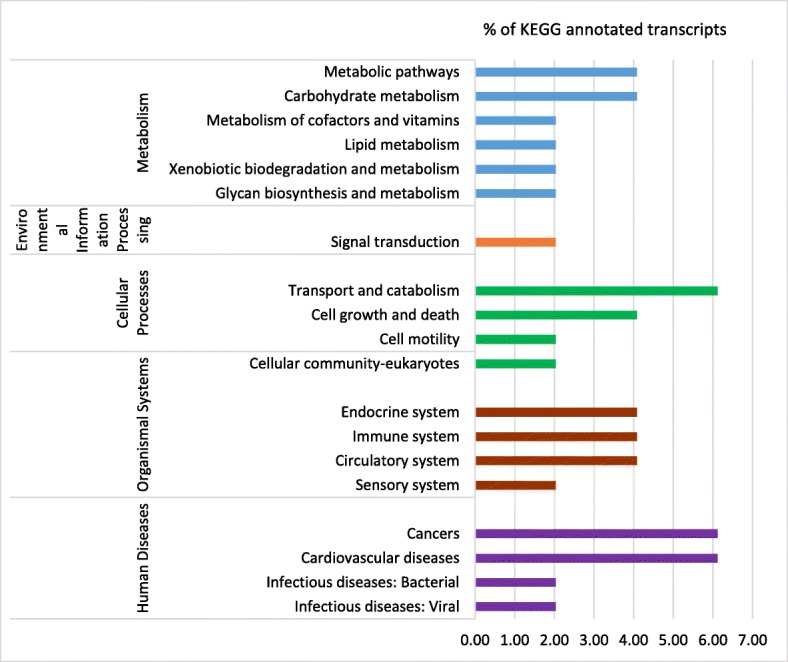
Summary of the KEGG reference pathways associated with up-regulated genes from whiteflies fed on CYSDV-infected melon (CYSDV whiteflies) for 7 days. Bars represent the percentage of the total KEGG annotated transcripts (12 genes out of a total of 49 up-regulated genes) in the CYSDV-whiteflies after 7 days feeding compared to virus-free whiteflies

KEGG (Kyoto Encyclopedia of Genes and Genomes) pathway analysis was performed on DEGs identified by the RNA-Seq experiments. This approach assists in the identification of potential pathways that were up-and down-regulated in whiteflies fed on CYSDV-infected melon at 72 h and 7 day feeding time points. Only 34% (28 out of 82) of the total number of genes up-regulated in CYSDV whiteflies at 72 h could be annotated using KEGG analysis [18]. Figure 2a provides a representation of the global functionality of the genes and summarizes the types of molecular pathways identified from up-regulated genes in CYSDV whiteflies at 72 h. The three categories of pathways most represented as up-regulated in whiteflies fed on CYSDV-infected melon for 72 h were 1) organismal systems, 2) human diseases, and 3) metabolism. Among significantly differentially expressed down-regulated genes at 72 h for CYSDV whiteflies, 29% (40 out of 139) were annotated to KEGG pathways, with the four most represented pathway categories identified as those associated with: 1) metabolism, 2) organismal systems, 3) human diseases, and 4) cellular processes (Fig. 2b). After the 7 day feeding period, 24.5% (12 out of 49) genes were annotated to KEGG pathways among those up-regulated in CYSDV whiteflies, with four categories accounting for most DEGs: 1) metabolic pathways, 2) human diseases, 3) cellular processes, and 4) organismal systems (Fig. 3). KEGG analysis was not performed for the 24 h or 7-day AAP down-regulated categories, because only three and two DEGs were present in CYSDV whiteflies, respectively. Detailed information about the genes, KEGG orthology (KO), annotations, score, and full pathways related to metabolism, genetic information processing, environmental information processing, cellular processes, organismal systems, and human diseases from CYSDV and VF whiteflies at 72 h and 7 days is available in Additional file 2. Gene categories that showed differences in the number of DEGs among the time points or those that showed the most differential expression are highlighted herein.

### Metabolism

Striking differences were observed within the metabolism category between CYSDV whiteflies after 72 h and 7 d feeding periods. A total of 15 metabolic pathway genes (four up-regulated and 11 down-regulated) were found to be differentially expressed in CYSDV whiteflies following the 72 h AAP, compared with only two metabolic pathway genes that were up-regulated in CYSDV whiteflies after the 7 day AAP (Additional file 2).

### Viral infectious diseases

Further analysis of KEGG data identified four up-regulated genes associated with the viral infectious disease category in CYSDV whiteflies. Three were identified after the 72 h AAP (*Bta03966*, DNA polymerase beta; *Bta00511*, nuclear transcription factor Y subunit beta; *Bta03569*, proteinral transcription factor IIE subunit) compared with only one (*Bta08780*, actin) at 7 days. In contrast, no DEGs related to virus infectious diseases were found to be down-regulated in CYSDV whiteflies compared to VF whiteflies for any of the three feeding periods (Additional file 2).

### Receptor

A single gene, *Bta00579*, encoding the orexin receptor type 2 also known as hypocretin receptor 2, was present among the up-regulated DEGs at 72 h. This protein is classified in the neuroactive ligand-receptor interaction category and is a G-protein coupled receptor involved in the control of sleep and arousal [19, 20]. No receptors were found among down-regulated genes at 72 h nor among any of the DEGs at 7 days in CYSDV whiteflies compared to VF whiteflies (Additional file 2).

### Signal transduction

Among genes associated with signal transduction pathways, five DEGs (three up-regulated- *Bta07544*, alkaline phosphatase; *Bta13636*, calmodulin; *Bta05793*, guanylate cyclase, and two down-regulated- *Bta013643*, tyrosine-protein kinase receptor, *Bta11166*, amino acid transporter, putative) were found in CYSDV whiteflies at 72 h compared with only one gene, *Bta08780*, actin (up-regulated) at 7 days (Additional file 2). Among the up-regulated signal transduction genes at 72 h, *Bta07544* is associated with the two-component system pathway while *Bta13636* is associated with many signal transduction pathways including Ras, Rap1, MAPK, apelin, calcium, phosphatidylinositiol, cAMP, and the cGMP-PKG signaling pathway. *Bta05793* is only known to be associated with the cGMP-PKG signaling pathway (Additional file 2). The two genes down-regulated at 72 h belonged to the MAPK signaling pathway (*Bta13643*) and the mTOR signaling pathway (*Bta11166*) (Additional file 2). The single up-regulated gene (*Bta08780*) identified after the 7 d AAP was associated with two signal transduction pathways: Rap1 and Hippo (Additional file 2).

### Transport and catabolism

A larger number of DEGs (nine genes) from transport and catabolism pathways were found in whiteflies given a 72 h AAP compared to those given a 7 day AAP, in which three different genes exhibited up-regulation. Up-regulated genes included those with functions associated with endocytosis/phagosomes (*Bta03727*, hepatocyte growth factor-regulated tyrosine kinase substrate), lysosomes/autophagy-animal (*Bta11419*, cathepsins B), and autophagy-yeast (*Bta09730*, proton-coupled amino acid transporter 4) were found in whiteflies given a 72 h AAP. In contrast, down-regulation was observed for six genes that belonged to five categories included those associated with phagosomes, lysosomes, peroxisomes, autophagy-animal, and autophagy-yeast (*Bta00173*, cathepsins L; *Bta03880*, cathepsins B; *Bta12604*, cathepsins B; *Bta10829*, acid phosphatase-1; *Bta14177*, fatty acyl-CoA reductase 1; *Bta17116*, Proton-coupled amino acid transporter 1). Following the 7 d feeding period, three different sets of genes belonging to phagosome, lysosome, and autophagy-animal categories (*Bta08780*, actin; *Bta08035*, cathepsin B; *Bta14774*, beta-hexosaminidase) were among those up-regulated in CYSDV whiteflies (Additional file 2).

### Immune system

CYSDV whiteflies were found to have a higher representation of DEGs associated with immune system related pathways compared with VF whiteflies. Six immunity genes (three up-regulated and three down-regulated) from three different immunity pathways were regulated differentially in CYSDV whiteflies after the 72 h AAP, whereas two genes from four immunity pathways exhibited reduced expression levels in CYSDV whiteflies after 7 days in comparison with expression levels in VF whiteflies (Additional file 2). Further analysis of those immunity related genes revealed five of the genes were cathepsins (four cathepsins B and one cathepsin L) that belonged to a NOD-like receptor signaling pathway and an antigen processing and presentation pathway (Table 3).

**Table 3:** Differential regulation of immunity genes in CYSDV whiteflies compared to virus-free (VF) whiteflies after feeding periods of 72 h or 7 days

Feeding time point	Gene ID	Annotation	FC (CYSDV/VF)	Immune pathway
72 h up-regulated genes	Bta11419	Cathepsin B	1.52	NOD-like receptor signaling pathway Antigen processing and presentation
	Bta00511	Nuclear transcription factor Y subunit beta	1.58	Antigen processing and presentation
	Bta05849	Cofilin/actin-depolymerizing factor-like protein	1.54	Fc gamma R-mediated phagocytosis
72 h down-regulated genes	Bta03880	Cathepsin B	0.64	NOD-like receptor signaling pathway Antigen processing and presentation
	Bta12604	Cathepsin B	0.45	NOD-like receptor signaling pathway Antigen processing and presentation
	Bta00173	Cathepsin L	0.56	Antigen processing and presentation
7 days up-regulated genes	Bta08780	Actin	1.78	Platelet activation Leukocyte transendothelial migration
	Bta08035	Cathepsin B	1.73	NOD-like receptor signaling pathway Antigen processing and presentation

### Common DEGs between CYSDV- and ToCV-whiteflies

**Fig. 4:**
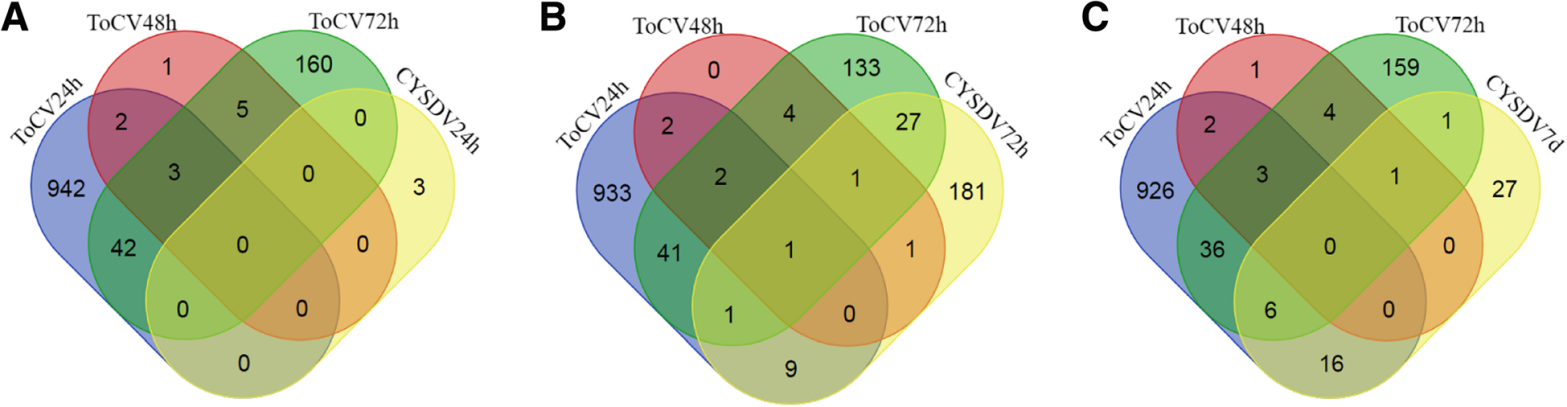
Venn-diagram showing unique and common DEGs in whitefly after feeding on ToCV-infected tomato plants for three different time periods (24 h, 48 h, and 72 h) or CYSDV-infected melon plants for three different time periods (24 h, 72 h, and 7 days). **a** Unique and common DEGs between ToCV whiteflies (24 h, 48 h, 72 h) and CYSDV whiteflies (24 h); **b** Unique and common DEGs between ToCV whiteflies (24 h, 48 h, 72 h) and CYSDV whiteflies (72 h); **c** Unique and common DEGs between ToCV whiteflies (24 h, 48 h, 72 h) and CYSDV whiteflies (7 days)

Of the 262 and 1155 unique DEGs found in CYSDV and ToCV-whiteflies, respectively, 59 were found to be differentially expressed in common between CYSDV-whiteflies and ToCV-whiteflies (Fig. 4). This included several major categories: 1) 20 orphan genes, 2) six genes associated with the lysosome including cathepsins (cathepsin B: *Bta12604*, *Bta11419*, *Bta08035*, *Bta03880*), cathepsin F-like protease (*Bta05911*), and one acid-phosphatase 1 (*Bta10829*), and 3) three genes were related to carbohydrate metabolism including one α-glucosidase (*Bta14422*), one sucrase (*Bta14312*) and one facilitated glucose transporter protein 1 (*Bta07749*) (Additional file 3). The cathepsin F-like protease, acid phosphatase-1, sucrase, and α-glucosidase genes showed the same trend of down-regulation in CYSDV- and ToCV-whiteflies compared to their expression in corresponding VF-whiteflies on melon and tomato plants, respectively, although at different feeding time points. The facilitated glucose transporter protein 1 gene (*Bta07749*) showed up-regulation in both CYSDV and ToCV whiteflies compared to VF whiteflies albeit at different feeding time points of 24 h and 72 h for ToCV and 7 days for CYSDV (Additional file 3). The 20 orphan genes and four cathepsin B genes did not show any definitive trend of regulation in CYSDV and ToCV whiteflies in comparison to VF whiteflies; however, the orphan gene category is a broad, relatively nondescript category and simply represents genes unique to the whitefly. Therefore, the category does not actually relate to gene functionality.

### Common DEGs among CYSDV-, ToCV-, and TYLCV-whiteflies

**Fig. 5:**
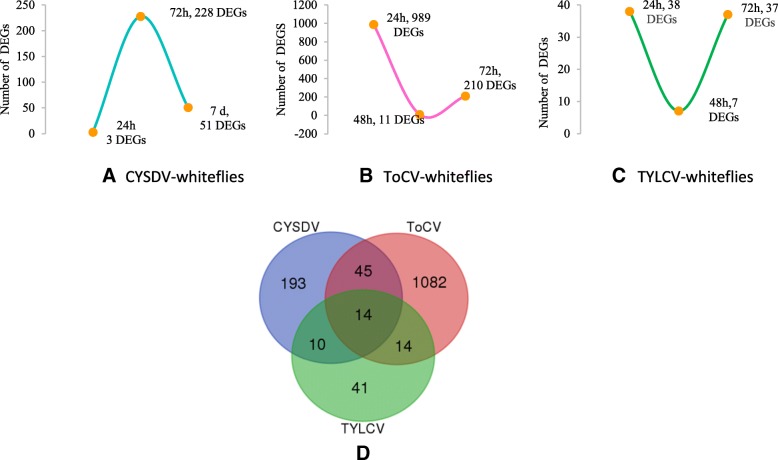
Trend of DEGs in CYSDV-whiteflies (**a**), ToCV-whiteflies (**b**), TYLCV-whiteflies (**c**) after feeding on host plants for three different time periods, **d**) Venn-diagram showing a unique and common DEGs among CYSDV-, ToCV-, and TYLCV-whiteflies, *B tabaci*, MEAM1

All DEGs identified from CYSDV-whiteflies, ToCV-whiteflies, and TYLCV-whiteflies compared to their respective virus-free controls were compared with one another in order to determine if there were any similar trends in temporal gene expression or if there were common DEGs. As noted above, previous studies identified 1155 DEGs in *B. tabaci* MEAM1 whiteflies that were provided three different AAPs of 24 h, 48 h, and 72 h on ToCV-infected tomato plants in comparison to whiteflies that fed on virus-free tomato [12]. Only 78 DEGs were found with *B. tabaci* MEAM1 after feeding on the same tomato cultivar infected with TYLCV in comparison to VF whiteflies [13] (Fig. 5). However, 28 of the DEGs were found to be common between ToCV- and TYLCV-whiteflies, even though the two viruses belong to two different genera (*Crinivirus* and *Begomovirus*) and have vastly different modes of transmission (Fig. 5d). Of these 28 DEGs, 58% showed the same pattern of either up- or down-regulation associated with feeding on tomato plants infected with either virus, while 42% showed opposite gene regulation patterns [13]. Fourteen of these genes showing differential expression between ToCV whiteflies and TYLCV whiteflies were also differentially expressed in whiteflies fed on CYSDV infected melon plants even though the whiteflies fed on a different host plant (Table 4; Fig. 5d). In the present study we investigated the transcriptional response in the whitefly after feeding on crinivirus infected melon plants using CYSDV, because unlike ToCV and TYLCV, CYSDV does not infect tomato. Such common DEGs among whiteflies fed on all three virus-host plants systems may indicate a universal reaction by the whitefly in response to feeding on plants infected by whitefly-transmitted viruses within the same genus (*Crinivirus*), or between the two genera differing in transmission mode (*Crinivirus* and *Begomovirus*), even though significant differences in expression did not always occur at the same time points or in the same manner (up- or -down regulation).

**Table 4:**
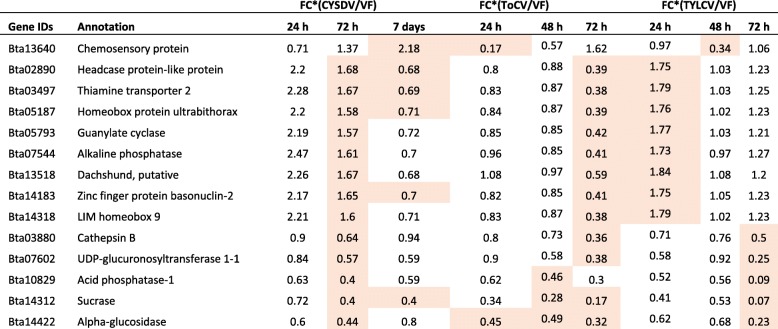
Common DEGs among CYSDV-whiteflies, ToCV-whiteflies, TYLCV-whiteflies in comparison to VF whiteflies when fed on CYSDV infected melon leaves for 24 h, 72 h, 7 days and on ToCV or TYLCV infected tomato leaves for 24 h, 48 h, 72 h

^*^Fold change values of CYSDV/ToCV/TYLCV whiteflies versus virus free whiteflies over three different feeding periods. Highlighted cells represent fold change values that differed significantly with FDR < 0.05

## Discussion

### DEGs in whiteflies associated with feeding on CYSDV-infected melon plants

Limited information is available with regard to the mechanism of how criniviruses associate with their whitefly vectors, although the location of this association is believed to be within the anterior foregut based on studies involving LIYV and *B. tabaci* NW (formerly Biotype A) [7–9]. Furthermore, knowledge of the whitefly response to the presence of a crinivirus in plant sap was limited to a recent study examining the influence of ToCV infection of its host plant on cellular gene expression changes associated with whitefly acquisition of the virus [12]. Although 275 DEGs were found in CYSDV whiteflies at three different feeding time points of 24 h, 72 h and 7 days, only three genes showed significantly different expression after the 24 h AAP, and all were down-regulated. In contrast, during the ToCV study in which gene expression was followed over a 72 h feeding period, nearly 1000 DEGs were identified within the first 24 h [12]. Very few differences were observed between treatments after the 48 h AAP, and 210 genes were differentially expressed after the 72 h AAP between ToCV-whiteflies and VF whiteflies [12]. Similarly, 221 DEGs were found at 72 h between CYSDV whiteflies and their corresponding VF whiteflies. Both virus-host-vector systems showed considerable differential expression at the last sampling point, although this time point differed between the two systems because the final sampling point focused on the number of days at which virus retention ceases after initial virus acquisition; three days for ToCV and seven days for CYSDV [3, 11].

### DEGs at the 72 h feeding time point

Two major categories among up-regulated genes following the 72 h AAP were orphan genes (21 DEGs) and genes encoding cuticle proteins (5 DEGs). Cuticle proteins are constituents of the cuticle, which is a highly organized structure produced as layered, extracellular secretions from the epidermis. The cuticle prevents xenobiotics and pathogens from entering insects and acts as a barrier to limit water loss. The expression of cuticle protein 1 was detected in the epidermis underlying the head and thorax (including legs and wings) of *Drosophila*, but not in the abdominal epidermis of newly eclosed whiteflies [21, 22]. Interestingly, the cuticle protein gene *MPCP4* of aphid, *Myzus persicae* was found to play a critical role in the acquisition of *Cucumber mosaic virus* (CMV; genus *Cucumovirus*, family Bromoviridae), a nonpersistently transmitted virus, as demonstrated by yeast-2-hybrid studies and RNA interference (RNAi) analysis [23]. The transcript levels of four cuticle protein genes (*MPCP1*, *MPCP2*, *MPCP4*, and *MPCP5*) were significantly higher in aphids after feeding on CMV-infected tobacco for 24 h. Yeast two-hybrid assays demonstrated the direct interaction of *MPCP4* protein with the CMV CP. The ability of *M. persicae* to acquire CMV was suppressed by RNAi against *MPCP4* [23]. Furthermore, In vitro studies have also shown a direct interaction of *M. persicae* MPCP2 and MPCP5 proteins with the helper-component protease (HC-Pro) of *Zucchini yellow mosaic virus* (genus *Potyvirus,* family Potyviridae), another virus that is transmitted in a non-persistent manner and whose virions associate with aphid stylets [24]. Although these last two examples represent different genera of nonpersistently transmitted viruses, the changes in expression of whitefly cuticle proteins after feeding on crinivirus-infected host plants warrants further study as these proteins may have an important role in acquisition and/or transmission of criniviruses.

A large number of orphan genes (54) were differentially regulated in CYSDV whiteflies at 72 h (21 up-regulated and 33 down-regulated) compared with those from VF whiteflies. Orphan genes are species-specific, and do not show any homology to the known genes of other organisms. Following the 7 d AAP, 20 orphan genes were up-regulated in CYSDV whiteflies (Additional file 4). Of the 74 differentially-expressed orphan genes between these two treatment time periods, five orphan genes (*Bta01566*, *Bta01754*, *Bta07683*, *Bta11224*, and *Bta15753*) showed common upregulation following both 72 h and 7 d feeding periods in CYSDV whiteflies compared to VF whiteflies. Gene structure analysis determined that 43 orphan genes out of a total of 69 unique orphan genes that were differentially regulated after either 72 h or 7 d AAPs in CYSDV whiteflies were short, with protein lengths of ≤200 amino acids. The structural organization of those orphan genes also revealed 20 of those orphan genes were located in clusters of two genes present on ten different scaffolds, while six orphan genes were present as clusters of three genes each on two different scaffolds (Additional file 4). Orphan genes constitute a large portion of every sequenced genome and are common among arthropods such as *Drosophila melanogaster* and *Aedes aegypti,* whose genomes contain 18.6 and 27.1% orphan genes, respectively [25–27]. In *D. pulex*, significant over-expression of tandemly duplicated orphan gene clusters were found when *D. pulex* was exposed to biotic and abiotic conditions [27]. Moreover, many orphan genes have been shown to be associated with species fitness and are under selection pressure in both *D. melanogaster* and mammals [28, 29]. Orphan genes have also been reported to encode surface antigens that are involved in host-parasite interactions in case of *Plasmodium* and *Theileria* [30]. In our previous study, we also found a large number of differentially expressed orphan genes, 337 out of a total of 1155 DEGs in whiteflies fed on ToCV infected tomato for periods of 24 h, 48 h, and 72 h compared to whiteflies fed on healthy tomato for the same time periods [12]. Interestingly 20 of the differentially expressed orphan genes found in whiteflies fed on ToCV infected and virus-free tomato were also found to be differentially expressed between whiteflies fed on CYSDV-infected and virus-free melon plants (Additional file 3). The presence of common differentially expressed orphan genes found in viruliferous whiteflies fed on two different host plants infected with two different criniviruses, indicates similarities in gene expression changes that occur at the cellular level in the vector when whiteflies feed on plants infected by criniviruses*,* and perhaps other semipersistently transmitted viruses.

The PEBP gene family is evolutionarily conserved and its functions are implicated in lipid binding, serine protease inhibition, and neuron development. Proteins encoded by this gene family have been shown to modulate several signaling pathways such as the MAP kinase pathway, NF-kappaB pathway, and glycogen synthase kinase-3 [31–34]. PEBPs were also found to be responsive to Mospilan insecticide in insecticide-resistant populations of *B. tabaci* MED (134 PEBP down-regulated) but were also down-regulated to a lesser degree in MED populations susceptible to Mospilan (16 PEBP down-regulated) [17]. The large expansion of the PEBP family that was found in *B. tabaci* MEAM1 suggests that these genes may be associated with insecticide resistance [17]. Interestingly, transgenic *D. melanogaster* overexpressing PEBP1 showed increased protection against bacteria due to release of immunity-related proteins in the hemolymph [35]. The expression level of PEBP protein was significantly downregulated in a *Bombyx mori* strain resistant to *Bombyx mori* nucleopolyhedrovirus (BmNPV) infection, suggesting involvement of PEBP proteins in the response of the resistant silkworm strain to BmNPV infection [36]. Significant down-regulation of 20 PEBPs genes in *B. tabaci* MEAM1 upon feeding on CYSDV infected melon plants may indicate a role for this gene family in the interaction of CYSDV with its vector, *B. tabaci*.

In addition to the 20 PEBPs, we also found 10 *AAA-ATPase domain containing protein genes* and four *Dynein* genes (belong to the AAA-ATPase family) down-regulated in CYSDV whiteflies compared to VF whiteflies after the 72 h feeding period. AAA-ATPases are associated with diverse cellular activities, such as DNA recombination, replication and repair, gene regulation, protein degradation, membrane fusion, peroxisome biogenesis, and intracellular transport [37–39]. Interestingly, baculovirus late expression factors, BmNPV LEF-11, involved in viral DNA replication and viral gene regulation were found to interact with ATPase family members ATAD3A and HSPD1 (HSP60) of *B. mori*, as demonstrated by co-immunoprecipitation (Co-IP) and mass spectrometry analyses [40]. Four genes containing AAA-ATPase domains were also found to be differentially regulated in ToCV whiteflies compared to VF whiteflies at 24 h and 72 h feeding time points [12]. Although criniviruses do not circulate or replicate within their whitefly vectors, it is clear that they influence the regulation of *B. tabaci* genes containing AAA-ATPase domains after whitefly vectors feed on crinivirus-infected plants.

### DEGs at 7 d feeding time point

Three major up-regulated gene categories were identified following the 7 d AAP, including 20 orphan genes, six cuticle proteins, and four myosin proteins (myosin regulatory light chain 2, myosin-7-like protein, myosin 2 light chain, and myosin-9, putative). Interestingly, the cuticle protein 1, *Bta14107*, up-regulated in CYSDV whiteflies following the 72 h and 7 d AAPs was also up-regulated in whiteflies when fed on ToCV-infected tomato for 24 h [12]. The timing of up-regulation clearly differed between the two systems, but the common expression supports a potentially important role for this cuticle protein, and likely others in whitefly acquisition and/or transmission of criniviruses.

Four myosins were upregulated specifically in CYSDV whiteflies at 72 h compared with VF whiteflies. Myosins are a large family of ATP-dependent motor proteins that function in muscle contractions and a wide range of motility processes in eukaryotes [41]. They are implicated in a number of critical cell functions, including cytokinesis, organelle transport and trafficking, and are also important in cell polarization [42] and signal transduction [43]. Herpesvirus virions engage myosin V for transport along the cortical actin network for fusion at the plasma membrane and are released into the synapse [44]. Expression of a dominant negative myosin Va protein in human cells reduced secretion of *Herpes simplex virus-1* (HSV-1) by 50–75% and simultaneously increased HSV-1 infectivity inside the cells, thus demonstrating myosin Va is involved in HSV-1 secretion from cells [45]. The myosin-actin interaction also plays an important role in releasing HIV-1 from host cells [46]. Myosins have also been found to be critical for cell-to-cell movement of tobacco mosaic virus in plants. Interference with the functions of three specific class VIII myosins and two class XI myosins significantly reduced the local and long-distance transport of the tobacco mosaic virus in tobacco [47]. It seems plausible that interactions with myosins may contribute to virus release from locations in the whitefly foregut.

### Interpretation of KEGG pathways analysis and identification of common patterns in whitefly gene expression associated with crinivirus transmission

KEGG Pathways Analysis identified several types of pathways within *B. tabaci* that are likely to be influenced by differential gene expression associated with CYSDV infection of melon host plants. Cathepsins are proteases that have wide biological implications including their involvement in protein degradation, apoptosis, as well as signaling, and their activity in the late endosome and lysosome has been widely implicated in virus transmission [48–50]. Five cathepsins (four cathepsins B and one cathespin L) associated with immunity related pathways were differentially expressed in CYSDV whiteflies, with some showing up-regulation and others reduced expression compared to virus free whiteflies. These cathepsins belonged to a NOD-like receptor signaling pathway and an antigen processing and presentation pathway (Table 3). Interestingly, 26 out of the total of 33 unique immune system related genes that were classified as belonging to an antigen processing and presentation pathway were also recently found to be differentially regulated in ToCV whiteflies compared with VF whiteflies [12], indicating a strong immune response by whiteflies to feeding on plants infected with either crinivirus (CYSDV or ToCV).

Cathepsins associated with transport and catabolism pathways were also found to be differentially regulated in CYSDV whiteflies compared with VF whiteflies after the 72 h AAP, and to a lesser degree the 7 d AAP. In our previous study involving ToCV, a large number of cathepsins (predominanty cathepsins B) associated with transport and catabolism pathways were differentially expressed in ToCV whiteflies compared to VF whiteflies [12]. Furthermore, in whiteflies fed on TYLCV-infected tomato plants for 72 h, four genes associated with the lysosome, three of which belonged to the cathepsin B family, as well as an acid phosphatase-1 gene, were differentially expressed compared to whiteflies fed on virus-free tomato [13]. Studies by Luan et al. [51] also revealed that genes associated with lysosome function were significantly up-regulated in TYLCCNV-whiteflies, but that study only examined a single time point [51]. The expression of a lysosomal cathepsin B gene was upregulated in the green peach aphid following acquisition of the *Potato leafroll virus* (PLRV), and further, the protein and virus were found to be co-localized in the cell membrane of the midgut [50]. We hypothesize that like other viruses including ToCV, TYLCV, and TYLCCNV, regulation of genes associated with the lysosome pathway, including the cathepsins, might be critical for CYSDV acquisition and transmission.

### Metabolism

Striking differences were observed within the metabolism category between CYSDV whiteflies after 72 h and 7 d feeding periods, with 11 genes downregulated after 72 h and only four up-regulated, whereas after 7 d only two DEGs, both downregulated, were identified in CYSDV whiteflies. An over-representation of metabolic pathway genes was also found in whiteflies fed on ToCV-infected tomato plants for 24 h and 72 h compared with those fed on virus free tomato plants [12]. This common pattern suggests that the over-representation of metabolic pathways may reflect changes in metabolic activity in response to whiteflies feeding on plants infected with criniviruses. For example, the downregulation of metabolic pathway genes in CYSDV whiteflies at 72 h may represent changes in metabolic requirements in response to feeding on the CYSDV infected melon plants, or even a reduction in metabolic activity associated with feeding on crinivirus infected plants compared to whiteflies that fed on healthy plants.

### Viral infectious diseases

KEGG Pathways Analysis identified four up-regulated genes associated with the viral infectious disease category in CYSDV whiteflies. No DEGs related to virus infectious diseases were found to be down-regulated in CYSDV whiteflies compared to VF whiteflies for any of the three feeding periods (Additional file 2). DNA polymerase beta (*Bta03966*) was one of three genes up-regulated after the 72 h AAP. It maintains genome integrity by participating in base excision repair required for DNA maintenance, replication, recombination, telomere processing, and drug resistance [52–54]. Overexpression of DNA polymerase beta mRNA has been correlated with a number of cancer types [55]. Human T-cell lymphotropic virus type I (HTLV-I) trans-activator protein, tax, is a trans-repressor of the human beta-polymerase gene [56]. Nuclear transcription factor (*Bta00511*), another gene up-regulated following the 72 h AAP, specifically recognizes a CCAAT box motif found in the promoters of its target genes and can function as both an activator and a repressor, depending on its interacting cofactors [57]. The third gene up-regulated after the 72 h AAP was *Bta03569,* which encodes a proteinral transcription factor IIE subunit [TFIIE] that plays a primary role in binding double-stranded DNA during transcription initiation [58]. Knockdown of TFIIE dramatically inhibited replication of the lentivirus HIV-I [59]. It is premature to predict how these upregulated genes may function in *B. tabaci* in association with feeding on crinivirus-infected plants, but their association with other virus infectious diseases warrants notation and potentially further study.

### Signal transduction

KEGG pathway analysis identified five DEGs associated with signal transduction pathways at 72 h. Among the up-regulated signal transduction genes at 72 h, *Bta07544* is associated with a two-component system pathway while *Bta13636* is associated with many signal transduction pathways including Ras, Rap1, MAPK, apelin, calcium, phosphatidylinositiol, cAMP, and the cGMP-PKG signaling pathway. *Bta05793* is only known to be associated with the cGMP-PKG signaling pathway. Only two DEGs associated with signal transduction were identified as downregulated at 72 h, with both belonging to the MAPK signaling pathway. Signal transduction, along with transport and catabolism pathways were the most represented classes of DEGs in ToCV whiteflies among down-regulated genes following the 24 h and 72 h AAPs, respectively in our previous study [12]. In that study 54 signal transduction genes associated with 20 KEGG pathways were down-regulated in whiteflies that fed on ToCV infected tomatoes compared with virus-free tomatoes. Those genes were associated with the phosphatidylinositol 3-kinase (PI3K)-Akt signaling pathway (13 genes), MAPK signaling pathway (17 genes), transforming growth factor-β (TGF-β) signaling pathway (three genes), Hippo signaling pathway (11 genes), and others [12]. A similar study examining interactions between *B. tabaci* and the begomovirus, *Tomato yellow leaf curl China virus* (TYLCCNV), also found that MAPK and TGF-β pathways were down-regulated [51]. This hints at the possibility of sensation signals in whitefly as a response to acquisition of CYSDV and perhaps other viruses.

### Common DEGs among CYSDV-, ToCV-, and TYLCV-whiteflies

A large number of differentially regulated orphan genes with high FPKM values were found in both CYSDV and ToCV whiteflies in comparison to their respective VF whiteflies, indicating these might be associated with virus-vector interactions. As discussed before, orphan genes comprised a significant portion of the sequenced genome of different species including humans, mosquito, and *Drosophila*, and they have also been implicated with species-specific adaptive processes, host-parasite interactions, and interactions with the environment [25, 60, 61]. The abundance of differentially expressed orphan genes in whiteflies associated with both crinivirus-host plant systems suggests these unique genes may also play an important role in crinivirus interactions with whitefly vectors.

It appears viruliferous whiteflies shut down their carbohydrate metabolism during acquisition of criniviruses by knocking down the expression of alpha-glucosidase and sucrase genes while increasing carbohydrate transportation activity by maintaining the higher expression of facilitated glucose transporter protein 1 gene in both ToCV and CYSDV systems. In addition, four genes related to carbohydrate metabolism, which included two α-glucosidases, one glycogen branching enzyme, and one sucrase, were also downregulated, with the exception of one α-glucosidase in TYLCV whiteflies compared to VF whiteflies [13]. Three genes associated with lysosome functions in the cathepsin B family also showed altered regulation in TYLCV whiteflies [13]. This suggests that carbohydrate metabolism and genes associated with lysosomes might be altered in viruliferous whiteflies when fed on host plants carrying each of three different viruses; CYSDV or ToCV (genus *Crinivirus*, family Closteroviridae) or TYLCV (genus *Begomovirus*, family Geminiviridae). Furthermore, such genes might encode functions critical for virus acquisition and transmission by the whitefly.

Whiteflies that fed on CYSDV infected melon plants compared with those fed on virus free melon plants exhibited patterns of differential expression overall that appeared to be delayed relative to patterns found in the ToCV-tomato study [12]. As noted previously, only minimal transcriptional differences were identified after 24 h between whiteflies feeding on CYSDV-infected melon and virus-free melon (3 DEGs), followed by much higher numbers of DEGs at 72 h (228 DEGs). Whiteflies were not sampled at 48 h in the CYSDV/melon system due to the observation during the previous studies of very few DEGs in whiteflies that fed on tomato plants with and without infection by ToCV and TYLCV at this time point [12, 13]. Because CYSDV is retained by the whitefly much longer (7 d) than ToCV (72 h), we examined gene expression at 7 d for whiteflies feeding on CYSDV-infected and virus-free melon plants and identified 51 DEGs (Fig. 5c). This lengthy AAP corresponds to the time point at which any whiteflies that acquired CYSDV within the first 24 h of feeding would lose their ability to transmit CYSDV based on previous studies [3].

Interestingly, whiteflies that fed on tomato infected with either ToCV or TYLCV showed similar trends in terms of the relative number of DEGs found at three different feeding time points (24 h, 48 h, and 72 h) (Fig. 5a & b) [13]. Higher numbers of transcriptional differences occurred between whiteflies fed on virus-infected tomato compared with those fed on virus-free tomato at 24 h (ToCV, 989 DEGs and TYLCV, 38 DEGs), followed by minimal transcriptional differences at 48 h (ToCV, 11 DEGs and TYLCV, 7 DEGs), and again higher numbers of DEGs at 72 h (ToCV, 210 DEGs and TYLCV, 37 DEGs), albeit a different set of genes showed differential expression at 72 h than was observed at 24 h for both viruses (Fig. 5a & b) [12, 13, 62]. This demonstrated a common response by *B. tabaci* MEAM1 to two viruses with very different modes of transmission as described by Hasegawa et al. [13]. ToCV and CYSDV are both semipersistently transmitted, and are believed to associate with the anterior foregut of the whitefly based on analogy to LIYV, another member of the *Crinivirus* genus [8, 9, 63], through a virus-encoded protein complex [7–9, 63], whereas TYLCV (Begomovirus) is persistently transmitted, becomes circulative within the insect vector, and is transmitted during egestion from the secondary salivary gland during feeding [10, 64–67].

A comparison of DEGs across the three different virus-host systems revealed 14 DEGs common among CYSDV-whiteflies, ToCV-whiteflies, and TYLCV-whiteflies (Fig. 5d) compared with their respective VF-whiteflies. Five out of the 14 common DEGs (*Bta03880*, cathepsins B; *Bta07602*, UDP-glucuronosyltransferase 1–1; *Bta10829*, acid phosphatese-1; *Bta14312*, sucrase; *Bta14422*, alpha-glucosidase) exhibited down-regulation in whiteflies fed on each of the three virus infected host combinations compared to those fed on their respective virus-free source plants (Table 4). Surprisingly, eight out of these 14 common DEGs (*Bta02890*, headcase protein-like protein; *Bta03497*, thiamine transporter 2; *Bta05187*, homeobox protein ultrabithorax; *Bta05793*, guanylate cyclase; *Bta07544*, alkaline phosphatase; *Bta13518*, dachshund, putative; *Bta14183*, zinc finger protein basonuclin-2, and *Bta14318*, LIM homeobox 9) showed similar trends of up-regulation in both CYSDV-whiteflies and TYLCV-whiteflies compared with whiteflies fed on respective virus-free plants; however, an opposite trend of down-regulation was observed in ToCV-whiteflies (Table 4). This may relate to differences in the physiological response or timing of such a response within the whitefly to feeding on each of the three virus-host systems, which may influence the differential expression of the same whitefly genes in association with feeding on the different viruses and host plants. Clarification of the timing of differences in up- and down-regulation of these common genes will require further, more extensive analyses with a greater number of time points.

## Conclusions

A total of 275 differentially expressed genes (DEGs) were identified in replicated experiments over three feeding periods between whiteflies fed on melon leaves with and without infection by the semipersistently transmitted crinivirus, CYSDV. Only 3 DEGs were identified following a 24 h AAP, sharply contrasting with results of experiments involving another crinivirus, ToCV on tomato, which exhibited 989 DEGs after a 24 h AAP using the same methodology. In contrast, the number of DEGs were comparable between whiteflies fed on each of the two virus-host systems at 72 h (210 DEGs with ToCV whiteflies on tomato, and 221 DEGs with CYSDV on melon). Following a 7 d AAP, 51 genes were found to be differentially expressed between CYSDV- and VF-whiteflies. Over the three sampling periods a total of 59 genes were found to be differentially expressed in common between *B. tabaci* MEAM1 whiteflies fed on host plants infected with either of the criniviruses, ToCV or CYSDV, compared with their respective virus free host plants. Similarly, 28 genes were differentially expressed in common between whiteflies fed on CYSDV infected melon and those that fed on tomato infected with the persistently transmitted begomovirus, TYLCV. Finally, 14 genes were differentially expressed in common among whiteflies fed on melon infected with the crinivirus CYSDV, tomato infected with the crinivirus ToCV, or tomato infected with the begomovirus, TYLCV. Of these, eight showed similar patterns of up-regulation in CYSDV-whiteflies and TYLCV-whiteflies, while surprisingly, down-regulation was observed in ToCV-whiteflies. Others shared common downregulation patterns. This suggests it may be possible to anticipate that certain changes in whitefly gene expression will occur when whitefly vectors feed on virus infected host plants compared to those that feed on virus-free host plants. These common whitefly gene expression changes may even occur when whiteflies feed on different host plants that are infected with viruses that have distinctly different modes of transmission. Some major categories of DEGs that were noteworthy due to prevalence and/or level of expression included orphan genes (broad category of unknown genes unique to the whitefly and not found in the genomes of other organisms), genes associated with the lysosome including cathepsins, genes encoding phosphatidylethanolamine-binding proteins (PEBP), and AAA-ATPase domain containing proteins.

### Methods

#### Whitefly feeding and RNA isolation

*Bemisia tabaci* MEAM1 from the Salinas USDA-ARS colony, originally collected from Imperial County, CA in 2013, were reared on broccoli (*Brassica oleracea*) plants. Whiteflies were provided acquisition access periods (AAPs) on CYSDV-infected or virus-free melon plants (cv. Topmark) for 24 h, 72 h, and 7 d. The presence of CYSDV in host plants and whiteflies was confirmed by RT-PCR from each feeding time point, and similarly virus-free plants and whiteflies were confirmed virus free by negative RT-PCR results as described in [5]. Following each AAP, whiteflies (200–400 per sample) were collected from CYSDV-infected or virus-free melon plants for each of the three biological replications, resulting in a total of 18 samples. Total RNA was extracted from each sample using TRIzol (Invitrogen, USA) followed by the Direct-zol RNA MiniPrep kit (Zymo Research Corporation, USA), according to instructions provided by the manufacturer. Whitefly titers were evaluated on extracts from two additional replications of 72 h and 7 days using RT-qPCR with primers and probes designed to the RNA-dependent RNA polymerase region of CYSDV RNA1 as described in [6].

#### Transcriptome sequencing and analysis

RNA extracted from whiteflies was used to construct RNA-Seq libraries using the protocol described in [68]. A HiSeq 2500 (Illumina, Inc. USA) was used for sequencing and RNA-Seq data was analyzed as described in Chen et al. [17]. RNA-Seq raw reads were processed and aligned to the MEAM1 reference genome. Raw counts were derived and then normalized to FPKM (fragments per kilobase of transcript per million mapped fragments). EdgeR was used to perform differential expression analysis. The following cutoff parameters were used for the identification of up- or down-regulated genes (adjusted *p* values < 005),: genes with FC ratio ≥ 1.5 for up-regulated genes and FC ratio ≤ 0.67 for down-regulated genes. More stringent criteria were used to identify individual genes in depth with FPKM > 10 and ratio ≥ 2 (up-regulated genes) and ratio ≤ 0.5 (down-regulated genes) [12]. KEGG annotations and pathways were generated using the deduced protein sequences of DEGs and the KEGG database [18]. Venn diagrams were created using webtool from Bioinformatics and Evolutionary Genomics, Ghent University [69].

## Additional files

Additional file 1: a. The number of Illumina raw and processed reads produced per RNA-Seq library from virus free (“VF”) whiteflies, *Bemisia tabaci* MEAM1, and whiteflies fed on cucurbit yellow stunting disorder virus (CYSDV) infected melon plants (“CYSDV”) for 24 h, 72 h, and 7 d. b. Correlation matrix analysis for multiple biological replicates obtained from RNA-Seq libraries prepared from virus free (VF) whiteflies fed on uninfected melon plants and whiteflies fed on cucurbit yellow stunting disorder virus (CYSDV) infected melon plants for periods of for 24 h, 72 h, and 7 d. c. Differentially expressed genes between virus free (VF) whiteflies fed on uninfected melon and whiteflies fed on cucurbit yellow stunting disorder virus (CYSDV) infected melon plants for 24 h, 72 h, and 7 d. (XLSX 33 kb)

Additional file 2: a. KEGG annotation, KO, and score values of the 28 genes out of a total of 82 up-regulated in CYSDV whiteflies versus virus-free whiteflies at 72 h. b. Pathway reconstruction results from the 28 genes out of a total of 82 up-regulated in CYSDV whiteflies versus virus-free whiteflies at 72 h. c. KEGG annotation, KO, and score values of the 40 genes out of a total of 139 down-regulated in CYSDV whiteflies versus virus-free whiteflies at 72 h. d. Pathway reconstruction results from the 40 genes out of a total of 139 down-regulated in CYSDV whiteflies versus virus-free whiteflies at 72 h. e. KEGG annotation, KO, and score values of the 12 genes out of a total of 49 up-regulated in CYSDV whiteflies versus virus-free whiteflies at 7 d. f. Pathway reconstruction results from the 12 genes out of a total of 49 up-regulated in CYSDV whiteflies versus virus-free whiteflies at 7 d. (XLSX 49 kb)

Additional file 3: A list of 59 common differentially expressed genes between ToCV whiteflies and CYSDV whiteflies compared to virus free whiteflies at all three different feeding time points. (XLSX 33144 kb)

Additional file 4: A list of 69 differentially regulated orphan genes found in CYSDV whiteflies compared to VF whiteflies following feeding for 72 h and 7 days. (XLSX 21 kb)

## Data Availability

The RNA-Seq data is available from the NCBI/GenBank BioProject database under SRA accession number: SRP163655.

## Abbreviations

AAP: Acquisition access period
CYSDV: Cucurbit yellow stunting disorder virus
CYSDV whiteflies: Whiteflies fed on CYSDV-infected melon plants
DEG: Differentially expressed gene
KEGG: Kyoto Encyclopedia of Genes and Genomes
LIYV: Lettuce infectious yellows virus
MED: *Bemisia tabaci* cryptic whitelfy species Mediterranean whitefly
MEAM1: *Bemisia tabaci* cryptic whitefly species Middle East Asia Minor 1
ToCV: Tomato chlorosis virus
ToCV whiteflies: whiteflies fed on ToCV-infected melon plants
TYLCCNV: Tomato yellow leaf curl China virus
TYLCV: Tomato yellow leaf curl virus
VF whiteflies: whiteflies fed on virus-free or uninfected plants
